# Correlation between Galectin-3 and Early Herpes Zoster Neuralgia and Postherpetic Neuralgia: A Retrospective Clinical Observation

**DOI:** 10.1155/2020/8730918

**Published:** 2020-04-14

**Authors:** Tingting Wang, Yong Fei, Ming Yao, Jiachun Tao, Jiajia Deng, Bing Huang

**Affiliations:** Department of Anesthesiology and Pain Medicine, The Affiliated Hospital of Jiaxing University, 1882 ZhongHuan South Road, 314001 Jiaxing, China

## Abstract

This study aims to explore the value of serum galectin-3 in patients with herpes zoster neuralgia (HZN) and postherpetic neuralgia (PHN) and other factors influencing HZN and PHN occurrence. Samples from forty patients with herpes zoster neuralgia (HZN) (Group H), 40 patients with nonherpes zoster neuralgia (Group N), and 20 cases of health check-up were collected. Patients were divided into PHN group (Group A) and non-PHN group (Group B) according to the occurrence of PHN in Group H. Galectin-3, T-lymphocyte subsets, and IL-6 were recorded in all patients. The changes of galectin-3 in patients with early HZN and PHN were analyzed by single-factor analysis and multifactor analysis. The age (*P*=0.012) and NRS scores (*P* < 0.001) of PHN patients were significantly higher than those of non-PHN patients and other neuralgia patients. The ratio of CD3+ (*F* = 80.336, *P* < 0.001), CD4+ (*F* = 12.459, *P* < 0.001) lymphocyte subsets, and CD4+/CD8+ (*F* = 15.311, *P* < 0.001) decreased significantly in PHN patients. The level of blood IL-6 (*F* = 139.446, *P* < 0.001) in PHN patients was significantly increased. Serum galectin-3 was significantly higher in HZN patients than in PHN patients (*P* < 0.05); IL-6 (OR = 10.002, 95% CI: 3.313–30.196, *P* < 0.001) and galectin-3 (OR = 3.719, 95% CI: 1.261–10.966, *P*=0.017) were the risk factors for HZN; galectin-3 (OR = 17.646, 95% CI: 2.795–111.428, *P*=0.002) was also the risk factor for PHN. ROC curve analysis also showed that serum galectin-3 was a better predictor of poor prognosis (AUC = 0.934, *P* < 0.001). Therefore, as an independent risk factor of HZN and PHN, serum galectin-3 may be used as a new biochemical marker in clinical practice.

## 1. Introduction

Herpes zoster (HZ) is an acute viral infection caused by varicella zoster virus (VZV). It is a common skin disease in clinical practice. Acute herpes zoster neuralgia (HZN) often occurs in the acute stage. Delayed and ineffective treatment of HZN may result in postherpetic neuralgia (PHN). Clinically, postherpetic neuralgia is defined as persistent severe pain in the area one month after recovery from shingles [1]. PHN is one of the most severe painful diseases affecting patients' quality of life. It occurs in patients over 60 years of age with herpes zoster. The incidence rate is about 50–75%, and the pain lasts for more than one year in about 10–25% of patients [2]. However, the pathogenesis of PHN has not been fully elucidated, and treatment is still a world problem. Galectin-3, a non-antibody protein, is a galactoside-binding animal lectin and a relatively new biomarker. Its biological function is to participate in acute, slow, and allergic inflammatory processes, and it is a powerful inflammatory response substance [3, 4]. However, the application of galectin-3 in HZN and PHN is rarely reported. This study aimed to compare the levels of serum galectin-3 in patients with HZN and PHN as well as to analyze the role of serum galectin-3 in patients with HZN and PHN.

## 2. Clinical Materials and Methods

### 2.1. Clinical Materials

The present study was approved by the Medical Ethics Committee of the First Hospital of Jiaxing (approval no. LS2019-218) and informed consent was provided by each participating patient. Samples from forty patients with herpes zoster neuralgia (Group H) and 40 patients with nonherpes zoster neuralgia (Group N) were collected upon patients' arrival for the initial visit from November 2017 to December 2019, including 16 cases in the PHN group and 24 cases in the non-PHN group. Nonherpes zoster neuralgia includes pleurisy and intercostal neuralgia. Samples from twenty healthy individuals who participated in a physical examination (Group C) were collected at the same time. A total of 100 patients were included, including 40 males and 60 females (average age 65.38 ± 1.14 years). The inclusive criteria are as follows: typical clinical manifestations, in line with the diagnostic criteria for herpes zoster; all were first-time visits; age 30 to 80 years old; patients who knew the purpose of the study and agreed to cooperate with the study and signed informed consent to participate in the study voluntarily. Missing patients were not included. The exclusion criteria are as follows: combined with other causes of nerve pain; self treatment or hospitals to give antiviral, nutritional nerve treatment; course of disease more than 10 days; combined with serious heart, lung, or kidney disease; patients with long-term use of hormones and immunosuppressive agents; patients with malignant tumors undergoing treatment; and patients with other contraindications for hormones.

### 2.2. Observation Indicators

General clinical data, including sex, age, and comorbidities, were recorded. Galectin-3, T-lymphocyte subsets subpopulation ratio and interleukin-6 (IL-6) were recorded in all patients. The first visit time, Numerical Rating Scale (NRS) score, and lesion area were recorded simultaneously in HZN patients. The size of the palm of the patient's hand was used to calculate the size of the rash. The palm area of the patient's hand was 1%. The size of the lesion was divided into <3% for small area, 3%–5% for medium area, and >5% for large area. The visual analog scale (VAS) was 0–10 cm, 1 cm represented 1 point, 0 point means painless, and 10 points means the most severe pain.

### 2.3. Specimen Collection

The blood samples were collected from the patients in our hospital and stored. The blood was centrifuged at 1000*g* for 5 minutes, and the serum was collected and stored in a refrigerator at 80°C. The levels of galectin-3, IL-6, and T-lymphocyte subsets (CD3+, CD4+, and CD8+) in serum were measured in all patients. Serum galectin-3 was tested with an ELISA kit manufactured by the American company eBioscience. Serum IL-6 was determined by electrochemiluminescence immunoassay (ECLIA) using the IL-6 detection kit (RD, USA). The cell percentages of T-lymphocyte subsets (CD3^+^, CD4^+^, and CD8^+^) were measured by flow cytometry (Becton, Dickinson and company, USA), and the CD4^+^/CD8^+^ was calculated. All operations were carried out in strict accordance with kit instructions.

### 2.4. Therapy

All patients in Group H were treated with conventional drug therapy and nerve block. Nerve block therapy: according to the area of the patient's herpetic lesion, the patients were given epidural catheters and connected with electronic analgesia pump (YBZ/China 1529–2015) or a peripheral nerve block. The prescription of epidural analgesia pump includes 0.75% ropivacaine hydrochloride 225 mg, 0.5 mg mecobalamin injection, 4 ml/h intravenous infusion, and 2 ml single additional dose, the locking time was 30 min, and the infusion dose was adjusted according to the patient's pain degree. Each patient was tested by two electronic pain pumps. The prescription of peripheral nerve block includes 2% lidocaine hydrochloride 20 mg, 0.75% ropivacaine hydrochloride 7.5 mg, and mecobalamin injection 0.5 mg, once every other day, up to 5 times. Conventional drug therapy: aciclovir 0.5 g (intravenously guttae, b.i.d.), mecobalamin 0.5 mg (oral administration, t.i.d.), gabapentin 0.3 g (oral administration, t.i.d.), et al. One month after the healing of herpes zoster lesions, if PHN was found, CT-guided selective nerve destruction was performed (selective nerve impulse radiofrequency operation was performed if herpes zoster occurred in the extremities).

### 2.5. Follow-Up

**Figure 1:**
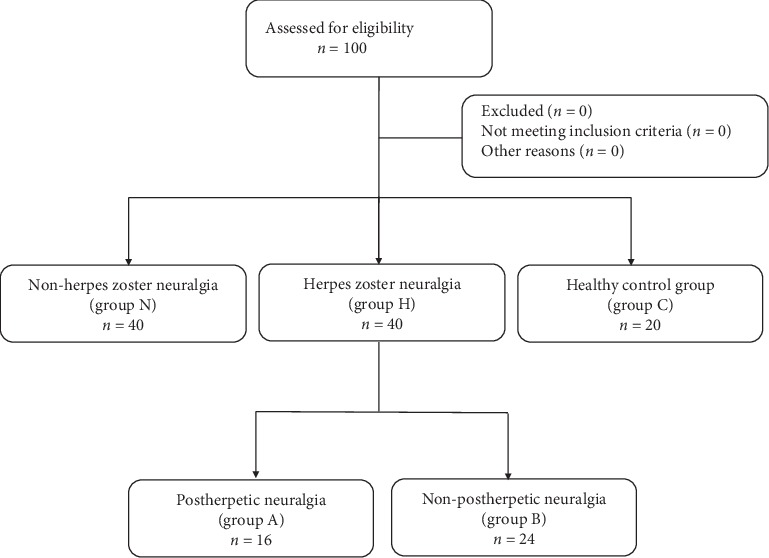
A flowchart of the clinical procedure.

After discharge, patients were followed up for 1 month, once a week. At one month, patients were divided into the PHN group (Group A) and non-PHN group (Group B) according to the occurrence of postherpetic neuralgia (Figure 1).

### 2.6. Statistical Analysis

SPSS 23.0 (SPSS Inc., Chicago, IL) was used to analyze the data. If the measurement data are in the normal distribution, it is expressed by (*X* ± *s*), *t*-test or analysis of variance (ANOVA) was used for the comparison between groups. The counting data were expressed by percentage or absolute value, and the ratio between the two groups was tested by the chi-square test. The relative factors of PHN were analyzed by a logistic regression model. A *p* value of <0.05 was considered the cut-off point for statistical significance.

## 3. Results

### 3.1. Comparison of General Clinical Data among Group A, Group B, and Group N

The results showed that the age of Group A was significantly higher than that of Group B and Group N (*P* < 0.05); The NRS score of Group A was significantly higher than that of Group B and Group N; the difference was statistically significant (*P* < 0.01). There were no significant differences among the three groups in sex, visiting time, skin lesion area, and disease complication (*P* > 0.05; Table 1).

### 3.2. Comparison of Lymphocyte, IL-6, and Galectin-3 in Different Groups of Patients

Comparison of the ratio of CD3+, CD4+, and CD8+ lymphocyte subsets and CD4+/CD8+ in H Group, N Group, and C group was performed. The results showed that the ratios of CD3+, CD4+ lymphocyte subsets, and CD4+/CD8+ in Group H and Group N were significantly lower than those in the normal group (*P* < 0.01). The results of multiple comparisons showed that the ratio of CD3+ (*F* = 80.336, *P* < 0.001), CD4+ (*F* = 12.459, *P* < 0.001) lymphocyte subsets, and CD4+/CD8+ (*F* = 15.311, *P* < 0.001) in Group H was significantly lower than that in Group N (*P* < 0.001). The ratios of CD8+ lymphocyte subsets in Group H and Group N were higher than that in Group C, but the differences were not statistically significant (*P* > 0.05).

### 3.3. Comparison of Serum IL-6 in Group H, Group N, and Group C

The results showed that the level of blood IL-6 (*F* = 139.446, *P* < 0.001) in Group H was the highest, followed by Group N and Group C, and the difference was statistically significant. The results of the multiple comparisons showed that the differences were statistically significant (*P* < 0.05).

### 3.4. Comparison of Serum Galectin-3 among Group H, Group N, and Group C

**Figure 2:**
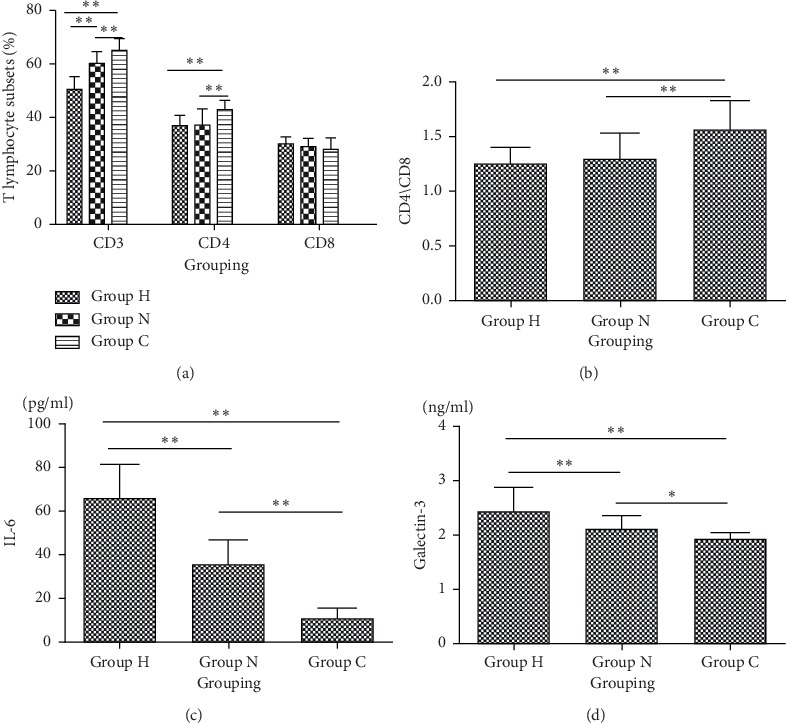
Comparison of (a, b) blood lymphocytes, (c) IL-6, and (d)galectin-3 among Group H, Group N, and Group C. Group H, patients with herpes zoster neuralgia; Group N, patients with nonherpes zoster neuralgia; Group C, healthy controls; IL, interleukin.

The results showed that serum galectin-3 was the highest in Group H, followed by N Group and C Group, and the difference was statistically significant (*P* < 0.05). The results of multiple comparisons showed that the differences were statistically significant (*P* < 0.05; Figure 2).

### 3.5. Comparison of the Ratio of Blood Lymphocyte, IL-6, and Galectin-3 between Group A and Group B

**Figure 3:**
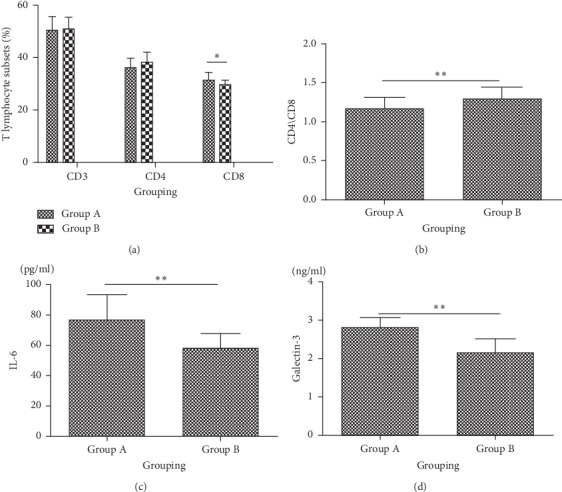
Comparison of the ratios of (a, b) blood lymphocytes, (c) IL-6, and (d) galectin-3 between Group A and Group B. Group A: patients with postherpetic neuralgia. Group B: patients without postherpetic neuralgia; IL, interleukin.

The ratios of CD3+, CD4+, CD8+, and CD4+/CD8+ in the blood of patients in Group A and Group B were compared. The results showed that there were significant differences in the ratio of CD8+ (*t* = −2.370, *P*=0.023) and CD4+/CD8+ lymphocyte subsets (*t* = 2.867, *P*=0.007) between the two groups, and the ratio of CD8+ lymphocyte subsets increased in Group A, while CD4+/CD8+ decreased in Group A compared with Group B. The level of IL-6 in Group A was significantly higher than that in Group B (*t* = −4.537, *P* < 0.001). The content of galectin-3 in blood in Group A was higher than that in Group B (*t* = −6.663, *P* < 0.001; Figure 3).

### 3.6. Multivariate Logistic Regression Analysis Affecting HZN (Group H)

Whether or not HZN occurs was used as the dependent variable (0 = neuralgia without HZN, 1 = HZN). The independent variables were meaningful, including age, sex, the period of the initial clinic visit, combined disease, NRS score, and percentage of CD8+, CD4+/CD8+, IL-6, and galectin-3. The median of patients with HZN and neuralgia without HZN of age, NRS score, percentage of CD8+, CD4+/CD8+, IL-6, and galectin-3 were 63.0, 5.0, 29.0, 1.25, 49, and 1.005, respectively. According to the median of the above continuous variables, they were divided into two groups: the Up Group and the Down Group. The results showed that IL-6 (OR = 10.002, 95% CI: 3.313–30.196, *P* < 0.001) and galectin-3 (OR = 3.719, 95%CI: 1.261–10.966, *P*=0.017) were the risk factors for HZN. The risk of HZN was 3.719 times higher in galectin-3 ≥1.005 ng/ml than in Galectin-3 <1.005 ng/ml (*P*=0.017; Table 2).

### 3.7. Multivariate Logistic Regression Analysis Affecting PHN (Group A)

Whether or not PHN occurs was used as the dependent variable *e* (0 = non-PHN, 1 = PHZ). The independent variables were meaningful, including age, sex, the period of the initial clinic visit, combined disease, NRS score, and percentage of CD8+, CD4+/CD8+, IL-6, and Galectin-3. The median of patients with PHZ and non-PHN of age, NRS score, percentage of CD8+, CD4+/CD8+, IL-6, and galectin-3 were 64.0, 5.0, 30.0, 1.25, 60.5, and 2.5, respectively. According to the median of the above continuous variables, they were divided into two groups: the Up Group and the Down Group. The results showed that galectin-3 (OR = 17.646, 95% CI: 2.795–111.428, *P*=0.002) was the risk factor for PHN. The risk of PHN was 17.646 times higher in galectin-3 ≥2.50 ng/ml than in Galectin-3 <2.5 ng/ml (*P*=0.002; Table 3).

### 3.8. Analysis of ROC Curve

**Figure 4:**
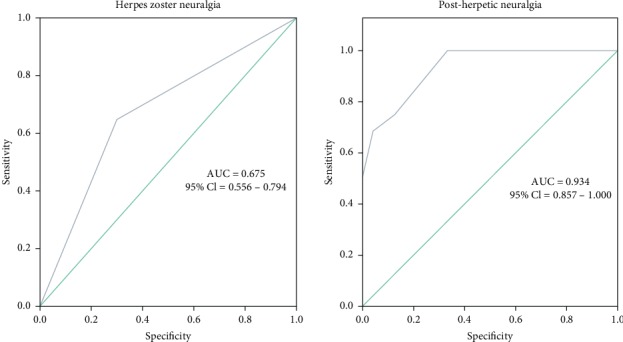
ROC curve distribution of galectin-3 for (a) HZN (Group H) and (b) PHN (Group A).

Whether or not HZN occurs was used as a state variable; the area under the ROC curve (AUC) of galectin-3 was calculated. The result showed that the area was 0.675 (*P*=0.007; Figure 4; Table 4), the best cut-off point of the ROC curve was 2.45 ng/ml, the sensitivity was 60.0%, and the specificity was 85.0%.

Whether or not PHN occurs was used as a state variable; the area under the ROC curve (AUC) of galectin-3 was calculated. The result showed that the area was 0.934 (*P* < 0.001; Figure 4; Table 5), the best cut-off point of the ROC curve was 2.45 ng/ml, the sensitivity was 99.9%, and the specificity was 66.7%.

## 4. Discussion

PHN is often a persistent burning pain, or intermittent shooting pain, and will be accompanied by a variety of sensory abnormalities or disappear. Sleep can not only temporarily reduce the intensity of pain, but emotional excitement, the increase in ambient temperature, and fatigue will increase the intensity of pain, severely hampering the patient's body function, but also reduce the quality of life of patients; the elderly are particularly serious. Until now, there has been no specific treatment for PHN, limited to antiviral therapy and symptomatic treatment of acute pain with analgesics. Single-factor analysis of this study found that age and NRS scores were significantly higher in PHN patients than in non-PHN patients, which was consistent with previous studies. The incidence of PHN increased with age [5, 6]. Acute herpetic pain is considered to be a risk factor for PHN. Therefore, effective control of acute herpetic pain for the prevention of PHN is of great significance [7].

IL-6 is a multieffect cytokine produced mainly by Th2 cells, monocytes/macrophages, and endothelial cells. It plays an important role in immune response, acute-phase reaction, and hematopoietic regulation and can activate the target gene. As a differentiation and growth factor of B cells, T cells, endothelial cells, etc., it participates in humoral immunity, induces megakaryocyte maturation, and the growth and differentiation of peripheral and central nervous system nerve cells. In this study, the serum levels of IL-6 in patients with PHN were significantly higher than those in non-PHN patients and healthy controls, suggesting that patients with high levels of IL-6 were more likely to develop PHN. IL-6 is associated with the development of PHN [8]. Previous studies have shown that cytokines, which are closely related to the activation and inflammation of the immune system, are involved in the formation of many acute and chronic neurodegenerative diseases. It is helpful to the normal development and repair of the nervous system in low expression condition, but it can damage the nervous system in high expression condition [9]. CD3+, CD4+, and CD4+/CD8+ in 16 patients with PHN were significantly lower than those in non-PHN and healthy controls, while CD8+ was significantly higher. Cassis et al. have shown that severe autoimmune disorders can be caused by a significant decrease in peripheral blood CD3+ and CD4+ [10], while immunosuppression can be intensified by a gradual increase in CD8+ levels [11, 12]. These results suggest that the decrease of T-lymphocyte subsets is closely related to the occurrence of PHN. Specific T-cell immune function can prevent virus activation, which plays a very important role in the elderly and immunocompromised people. Asanuma et al. found that the increased risk of PHN in older adults was also due to reduced serum VZV-specific CD4+ lymphocyte. This is due to the fact that CD4+ lymphocyte subsets expression decreases with age [13]. Studies have shown that patients with CD4+ and CD8+ deficiency are at high risk of herpes zoster virus infection, and the risk of developing PHN is relatively high [14].

Galectin-3 is a member of the galectin-binding lectin family [15] and is a multifunctional protein with a variety of biological functions, including tumor cell proliferation and invasion and angiogenesis [16–18]. The human gene is encoded by a single gene, LGALS3, with 251 amino acid residues and a relative molecular weight of 31KD. According to its chemical structure, it can be divided into three types: archetype, chimeric type, and tandem type. Of the 15 galectins identified so far, galectin-3 is the only chimeric type, which is usually expressed in a variety of cell types such as endothelial cells, epithelial cells, activated microglial cells, inflammatory cells (mainly macrophages), and various tissues and organs, including the spleen, stomach, colon, liver, kidney, heart, uterus, ovary, and pancreas [19]. As a multifunctional protein, galectin-3 is involved in various physiological and pathological processes and plays a key role in the development of acute and chronic diseases, especially in cell growth, angiogenesis, carcinogenesis, and triggering of acute inflammation. In some chronic diseases, such as autoimmune diseases, arteriosclerosis, heart failure, diabetes, and other diseases in the development process are also involved [20]. Recent studies have shown that galectin-3 participates in the inflammatory response of microglia after ischemic injury, and its expression is upregulated [21, 22]. Galectin-3 is a powerful inflammatory signal, active in all stages of the inflammatory response. Its proinflammatory effects are shown in the following aspects: regulation of cell adhesion, promotion of cell activation and chemotaxis, and regulation of cell growth and apoptosis [23]. In this study, the levels of galectin-3 in patients with herpetic neuralgia were significantly higher than those in non-PHN patients. Logistic regression analysis showed that galectin-3 was an important risk factor for PHN (OR = 17.646, 95% CI: 2.795–111.428, *P*=0.002). The ROC curve analysis also showed that galectin-3 had a significant predictive effect on PHN (AUC = 0.934, *P* < 0.001). This suggests that galectin-3 may be a new biochemical marker for PHN occurrence.

The mechanism by which galectin-3 induces postherpetic neuralgia is unclear. It was found that galectin-3 was expressed in F4/80 and Iba1-positive cells of the spinal dorsal horn, suggesting that these cells secrete galectin-3 [24]. Galectin-3 has been reported to promote the expression of extracellular signal-regulated kinase 1/2 (ERK 1/2) mRNA and phosphorylation [25, 26]. In the neurons of the spinal dorsal horn, the ERK 1/2 pathway was activated in the neuropathic pain state [27], and galectin-3 activated the ERK pathway [28]. Therefore, the activation of ERK1/2 by galectin-3 may be involved in the induction of herpetic pain. Since there is increasing evidence that activated microglia release factors also play an important role in the development of neuropathic pain [29], another possible mechanism is involved in galectin-3's effect on microglia.

Our study has several limitations, the most important of which is the retrospective design of our research, and thus, selection and recall biases could not be avoided. Moreover, our results should be interpreted with caution due to the small sample size of our population, which limits the generalizability of our findings. Therefore, future prospective studies with larger sample sizes and longer follow-up durations are still warranted to validate our observations.

## 5. Conclusion

In conclusion, the study of IL-6 and lymphocyte subsets in HZN and PHN patients, especially galectin-3, is of clinical significance in the treatment of patients with HZN and PHN. This study further confirmed the important value of galectin-3 in HZN and PHN diseases. Therefore, it is reasonable to speculate that galectin-3 may be used as a biochemical marker in patients with HZN and PHN, and that interfering with the pathway of galectin-3 may be a potential therapeutic target.

## Figures and Tables

**Table 1:** Comparison of general clinical data of patients.

Variable	Herpes zoster neuralgia (*N* = 40)		Neuralgia without HZV (*N* = 40)	*F*/*χ*2	*P*
	PHN (*N* = 16)	Non-PHN (*N* = 24)			
Age	69.25 ± 8.87 a	62.13 ± 8.73 b	61.60 ± 8.70 b	4.683	0.012
Sex					
Male	6 (37.5) a	12 (50.0) a	19 (47.5) a	0.654	0.721
Female	10 (62.5) a	12 (50.0) a	21 (52.50) a		
NRS score	5.75 ± 1.13 a	4.63 ± 0.58 b	4.90 ± 0.74 b	10.189	<0.001
The period of initial clinical visit					
<1 d	2 (12.5) a	6 (25.0) a	9 (22.5) a	1.384	0.847
1∼3 d	7 (43.8) a	9 (37.5) a	18 (45.0) a		
>3 d	7 (43.8) a	9 (37.5) a	13 (32.5) a		
The surface area of skin					
<3%	6 (37.5) a	7 (29.2) a	16 (40.0) a	1.612	0.807
3–5%	5 (31.3) a	10 (41.7) a	16 (40.0) a		
>5%	5 (31.3) a	7 (29.2) a	8 (20.0) a		
Combined disease					
Yes	14 (87.5) a	19 (79.2) a	27 (67.5) a	2.756	0.252
No	2 (12.5) a	5 (20.8) a	13 (32.5) a		

PHN, postherpetic neuralgia; HZV, herpes zoster virus; *P*, *p* value; *N*, number; NRS, numerical rating scale; d, day. ^*∗*^Categorical variables are presented as numbers and percentages, while continuous variables are presented as mean ± standard deviation.

**Table 2:** Multivariate logistic regression analysis affecting HZN.

Variable	B	S.E.	Wald	Exp (B)	95% CI		*P*
					Lower limit	Upper limit	
Age	0.829	0.755	1.204	2.291	0.521	10.069	0.272
Sex	−0.642	0.710	0.818	0.526	0.131	2.116	0.366
The period of initial clinic visit	−0.0352	0.448	0.617	0.703	0.292	1.692	0.432
Combined disease	0.505	0.881	0.329	1.657	0.295	9.312	0.566
NRS	−0.044	0.839	0.003	0.957	0.185	4.960	0.958
CD8+	2.037	0.816	6.237	7.667	1.550	37.923	0.071
CD4+/CD8+	2.128	0.835	6.500	8.397	1.636	43.109	0.338
IL-6	2.303	0.564	16.685	10.002	3.313	30.196	<0.001
Galectin-3	1.313	0.552	5.667	3.719	1.261	10.966	0.017

NRS, numerical rating score; IL, interleukin; B, standardized coefficient; CI, confidence interval; HZN, herpes zoster neuralgia.

**Table 3:** Multivariate logistic regression analysis of PHN.

Variable	B	S.E.	Wald	Exp (B)	95% CI		*P*
					Lower limit	Upper limit	
Age	1.569	1.231	1.625	4.803	0.430	53.626	0.202
Sex	−1.346	1.293	1.084	0.260	0.021	3.280	0.298
The period of initial clinic visit	0.467	0.862	0.293	1.595	0.294	8.650	0.588
Combined disease	−1.057	1.424	0.551	0.348	0.021	5.663	0.458
NRS score	4.103	2.485	2.727	60.532	0.464	7888.792	0.099
Percentage of CD8+	0.472	1.144	0.170	1.604	0.170	15.112	0.680
CD4+/CD8+	−2.160	1.425	2.299	0.115	0.007	1.881	0.129
IL-6	1.597	1.252	1.626	4.937	0.424	57.438	0.202
Galectin-3	2.871	0.940	9.321	17.646	2.795	111.428	0.002

PHN, postherpetic neuralgia; NRS,: numerical rating score; IL, interleukin; B, standardized coefficient; CI, confidence interval; *P*, *p* value; HZN, herpes zoster neuralgia.

**Table 4:** ROC curve distribution of galectin-3 for HZN.

Variable	AUC	S.E.	95% CI		*P*
			Lower limit	Upper limit	
Galectin-3	0.675	0.061	0.556	0.794	0.007

ROC, receiver operating characteristic; HZN, herpes zoster neuralgia; CI, confidence interval; *P*, *p* value; AUC, area under curve. ^*∗*^*p* value of below 0.05 was considered the cut-off point of statistical significance.

**Table 5:** ROC curve distribution of galectin-3 for PHN.

Variable	AUC	S.E.	95% CI		*P*
			Lower limit	Upper limit	
Galectin-3	0.934	0.036	0.857	1.000	<0.001

ROC, receiver operating characteristic; PHN, postherpetic neuralgia; CI, confidence interval; *P*, *p* value; AUC, area under curve. ^*∗*^*p* value of below 0.05 was considered the cut-off point of statistical significance.

## Data Availability

The data used to support the findings of this study are included within the article.
